# Integrated management of atrial fibrillation and comorbidities in the community: a generalist-specialist collaborative RCT and subgroup analysis

**DOI:** 10.3389/fcvm.2026.1840644

**Published:** 2026-07-13

**Authors:** Dai Huimin, Bo Jun, Jiang Meng, Zhou Lulu, Huang Qian, Ying Xiaoying

**Affiliations:** 1 Weifang Community Health Service Center, Shanghai, China; 2 Department of Cardiology, Renji Hospital, Shanghai Jiao Tong University School of Medicine, Shanghai, China

**Keywords:** atrial fibrillation, cardio-cerebrovascular co-management, co-management of risk factors, community-based integrated management, generalist-specialist collaboration

## Abstract

**Objective:**

To develop, implement, and evaluate a community-based, patient-centered integrated care model for atrial fibrillation (AF) characterized by generalist-specialist collaboration and cardio-cerebrovascular co-management, and to examine whether its effectiveness varies across key patient subgroups.

**Methods:**

A prospective, single-blind, randomized controlled trial was conducted. Between June 2024 and May 2025, 160 patients with AF were consecutively enrolled from the Shanghai Weifang Community Health Service Center and Renji Hospital, affiliated to Shanghai Jiao Tong University, School of Medicine. Participants were randomly assigned to either a control group or an intervention group, with 80 patients in each. The intervention group received comprehensive management based on a collaborative framework between general practitioners (GPs) and specialists. The control group received routine community-based chronic disease management and follow-up. After a 12-month intervention period, outcomes were compared between the two groups, including the attainment rates for body mass index (BMI), blood pressure, and blood glucose control; levels of N-terminal pro-B-type natriuretic peptide (NT-proBNP); left ventricular ejection fraction (LVEF); standardized anticoagulation and heart rate control medication use; and the primary composite endpoint (heart failure or stroke). Subgroup analyses were prespecified for age (<75 vs. ≥75 years), sex, and CHA_2_DS_2_-VASc score (2–3 vs. ≥4). Interaction *P* values were derived from logistic regression models including treatment-by-subgroup product terms.

**Results:**

A total of 150 patients completed the 12-month follow-up, with 75 patients in each group. The intervention group demonstrated significantly higher attainment rates for BMI, blood pressure, and blood glucose control compared to the control group. Regarding cardiac function, LVEF showed significant improvement in the intervention group, whereas no significant difference was observed in NT-proBNP levels between the groups. Furthermore, the standardized usage rates for both anticoagulant and heart rate control medications were significantly higher in the intervention group. For primary endpoint events, the incidence of the composite outcome (heart failure or stroke) was significantly lower in the intervention group. Subgroup analyses revealed no significant interactions for age (*P* = 0.82), sex (*P* = 0.75), or CHA_2_DS_2_-VASc score (*P* = 0.18), indicating consistent intervention effects across these strata.

**Conclusion:**

The generalist-specialist collaborative integrated care model effectively improves risk factor control, cardiac function, and treatment standardization, and reduces major adverse cardiovascular events in community-dwelling AF patients. The benefits are consistent across age, sex, and comorbidity burden subgroups, supporting its broad applicability in primary care settings.

## 1 Background

Atrial fibrillation (AF) is the principal cause of cardiogenic stroke, accounting for approximately 70% of all such events. Mortality among patients with cardiogenic stroke are significantly higher than for non-cardiogenic stroke. In China, over 20 million individuals live with AF, and prevalence increases markedly with age (1, 2). In super-aged cities like Shanghai, where the proportion of elderly residents reached 37.6% in 2024 and is projected to exceed 40% by 2025 (3, 4), the disease burden of AF and the associated risk of cardio-cerebrovascular comorbidities are expected to grow exponentially (5).

Approximately one-third of AF patients are asymptomatic, with stroke often serving as the initial clinical manifestation. However, community-based AF screening rates remain below 15%, and the rate of missed diagnoses is as high as 60%. Under the current health system, fragmented management (6) between hospital-based specialists and primary care often results in information silos, redundant testing, ineffective referrals, and poor continuity of care. This contributes to inadequate risk factor control and low adherence to anticoagulant therapy, significantly increasing stroke risk. Recent guidelines and evidence (3, 7–10) highlight the need for integrated, patient-centered management of AF, emphasizing not only rhythm and rate control but also the comprehensive management of hypertension, diabetes, thyroid dysfunction, obesity, lifestyle factors, and other cardiovascular risk factors. Community-based collaborative care models that involve both general practitioners and specialists, supported by multidisciplinary teams and digital health platforms (11), have shown promise in improving risk factor control, patient adherence, and cardiovascular outcomes.

Based on previous research (12–14), the present study adopts an integrated, multidisciplinary, community-based AF management model led collaboratively by specialists and general practitioners, with participation from health assistants and family members. The aim is to optimize resource allocation and multifactorial intervention, and to empirically evaluate the effectiveness of such a model for improving quality of life and clinical outcomes in AF patients, thereby providing an evidence-based reference for community management strategies.

## 2 Subjects and methods

### 2.1 Study population

This study employed a prospective, single-blind, randomized controlled design. Between June 2024 and May 2025, 160 patients with atrial fibrillation (AF) who attended the Weifang Community Health Service Center in Pudong New Area were consecutively enrolled.

Sample Size Justification: The sample size was determined based on the primary composite endpoint (incidence of stroke, heart failure hospitalization, or all-cause mortality). Drawing on prior community-based AF management studies (15) reporting event rates of approximately 15%–20% in usual care groups, we anticipated a clinically meaningful absolute risk reduction of 10 percentage points with the integrated intervention. Using a two-sided significance level of 0.05% and 80% power, the minimum required sample size was estimated at 72 patients per group. Accounting for an anticipated 10% attrition rate, we enrolled 80 patients per group (160 total).

#### 2.1.1 Randomization

All eligible participants were randomly allocated to either a control group (*n* = 80) or an intervention group (*n* = 80) using a random number table to ensure balanced allocation.

**Inclusion criteria**: (1) AF diagnosed by 12-lead electrocardiogram (ECG) or 24 h Holter monitoring; (2) Non-valvular AF confirmed by echocardiogram, with AF duration exceeding one year; (3) Clear consciousness and the capacity for normal communication; (4) Willingness to participate in follow-up, residence within the Weifang Community, and voluntary provision of contact information.

**Exclusion criteria**: (1) Patients lost to follow-up or who relocated. (2) Individuals with mental disorders or poor compliance for any reason. (3) Patients with severe hepatic or renal insufficiency, or active malignancy. (4) Concurrent participation in any other relevant clinical trial.

**Ethical Considerations**: All enrolled patients provided written informed consent prior to study inclusion.

## 3 Study methods

### 3.1 Data collection

Patient health information was collected by the same researcher one day prior to enrollment and again at the 12-month follow-up. The collected data comprised:

(1) Demographic and Lifestyle Information: Age, gender, marital status, educational level, smoking status, alcohol consumption, dietary habits, physical activity, and sleep patterns.
(2) Medical History: Pre-existing conditions (e.g., hypertension, diabetes, coronary heart disease, stroke, dyslipidemia, heart failure); other comorbidities; family history of cardiovascular or cerebrovascular diseases; and psychological evaluation for symptoms of depression and anxiety.
(3) Physical and Laboratory Examinations: Height, weight, blood pressure, serum biomarkers, and relevant laboratory results. Baseline data were derived from measurements taken one day before enrollment.

### 3.2 Group allocation and management protocols

Both groups underwent comprehensive assessment and management of multiple cardiovascular and metabolic risk factors, including hypertension, diabetes, dyslipidemia, obesity, and lifestyle behaviors.

**Control group**: Management adhered to standard chronic disease protocols, primarily led by general practitioners. Outpatient health education and periodic telephone follow-ups were conducted. General practitioners routinely monitored and addressed major risk factors, provided guidance on lifestyle modification, and ensured regular assessment of blood pressure, blood glucose, and lipid profiles. Patient data were routinely recorded, but no targeted or specialized interventions beyond standard care were implemented.

**Intervention group**: Patients received multidisciplinary, community-based integrated management coordinated by cardiologists from Renji Hospital, general practitioners from Weifang Community, and specialized healthcare assistants. The key components of the management protocol were as follows:

(1) **Establishment of AF Specialty Outpatient Clinics**: Dedicated AF clinics were established within the community health center. General practitioners managed patient reception, while diagnostic and therapeutic strategies were jointly formulated by cardiologists and GPs. Healthcare assistants provided education to patients and their families regarding AF, stroke risks, and early recognition of stroke symptoms (e.g., using the FAST principle: Facial drooping, Arm weakness, Speech difficulties, Time to call emergency services). They also offered guidance on anticoagulant medication use. Multifactorial risk management was emphasized, with the team collaboratively monitoring and intervening for hypertension, diabetes, dyslipidemia, obesity, and unhealthy behaviors. Patients and caregivers were trained to self-monitor blood pressure, pulse, and body weight, and were encouraged to modify unhealthy lifestyles and control risk factors. Assistants facilitated data collection, ensured adherence to standardized measurement protocols, and supported the GPs' comprehensive management efforts.
(2) **Tiered Diagnosis and Referral System**: AF patients were stratified according to stroke risk (CHA_2_DS_2_-VASc score) and the need for procedural intervention or anticoagulation therapy. Low-risk patients (CHA_2_DS_2_-VASc score of 0 for males or 1 for females) did not receive anticoagulant therapy and were managed through routine follow-up by GPs at the community center. High-risk patients (CHA_2_DS_2_-VASc score ≥1 for males or ≥2 for females, or HAS-BLED score ≥3 indicating high bleeding risk) underwent more frequent clinical assessments and follow-up. Special populations with comorbidities, such as coronary heart disease or chronic kidney or liver dysfunction, were referred to a dedicated AF-cardiogenic stroke specialty clinic within the “Renji-Weifang” collaborative network. The referral pathway included routine referrals for newly diagnosed AF requiring rhythm control or for patients eligible for interventional or surgical management (e.g., catheter ablation, left atrial appendage occlusion), and emergency referrals for acute thromboembolic events, severe cardiovascular complications, or moderate-to-severe bleeding. Upon stabilization, patients were transitioned back to community-based follow-up.
(3) **Homogenized Medication Supply and Laboratory Testing**: The Weifang Community Health Service Center was equipped with novel oral anticoagulants, such as rivaroxaban. An extended prescription system facilitated access to common anticoagulants, including dabigatran, edoxaban, and warfarin, ensuring medication consistency across community and tertiary hospital settings. Essential laboratory tests (NT-proBNP, echocardiography, 24-hour Holter ECG, INR) were available at the community center, enabling seamless monitoring and management of AF patients across all levels of care.
(4) **Structured Telephone Follow-up**: A regular telephone follow-up schedule was implemented to monitor disease progression and reinforce patient education: weekly during the first month, biweekly in the second month, and monthly thereafter. These calls served to remind patients of medication adherence and self-monitoring, reinforce educational messages, and allow for timely treatment plan adjustments based on patient-reported outcomes and clinical indicators.

## 4 Statistical analysis

All statistical analyses were performed using SPSS version 21.0 software. Continuous variables with a normal distribution were presented as mean ± standard deviation $\left(\bar{x}\pm s\right)$. Between-group comparisons were conducted using independent samples *t*-tests, while within-group comparisons (pre- vs. post-intervention) were analyzed using paired samples *t*-tests. Non-normally distributed continuous variables were expressed as medians (M) and compared between groups using the Mann–Whitney *U* test. Categorical variables were presented as frequencies and percentages and analyzed using the chi-square (*χ*^2^) test. A *p*-value of less than 0.05 (*P* < 0.05) was considered statistically significant.

Subgroup analyses were prespecified for age (<75 vs. ≥75 years), sex, and CHA_2_DS_2_-VASc score [2–3 vs. ≥4 points (16)]. For each subgroup, event rates and odds ratios (ORs) with 95% confidence intervals (CIs) were calculated. Interaction *P* values were derived from binary logistic regression models including treatment, subgroup, and their product term. No adjustment for multiple comparisons was applied given the exploratory nature of the analyses.

## 5 Results

### 5.1 Comparison of baseline characteristics between groups

Table 1 shows of the 80 patients initially enrolled in the intervention group, 2 were lost to follow-up and 3 withdrew; similarly, of the 80 in the control group, 4 were lost to follow-up and 1 withdrew. Ultimately, 75 patients in each group completed the study. Baseline demographic and clinical characteristics—including age, gender, marital status, educational level, and comorbidities—were comparable between the two groups (all *P* > 0.05), indicating successful randomization and minimizing selection bias.

**Table 1:** Baseline characteristics of patients in the control and intervention groups.

Parameter	Control group (*n* = 75)	Intervention group (*n* = 75)	*t*/*χ*^2^/*Z*	*P*-value
Age (years)	78.14 ± 8.97	77.57 ± 8.88	0.885	0.376
Gender (*n*)			0.091	0.763
Male	37	35		
Female	38	40		
Marital Status (*n*)			0.15	0.698
Married	44	48		
Single	31	27		
Education Level (*n*)			1.672	0.643
Primary or below	34	40		
Junior high	24	17		
High school	10	11		
College or above	7	7		
Comorbidities (*n*)			0.022	0.883
Hypertension	62	69		
Diabetes	29	34		
Dyslipidemia	37	40		

### 5.2 Follow-up results of BMI, blood pressure, and blood glucose control rates

Table 2 shows at baseline, the control rates for BMI, blood pressure, and blood glucose were comparable between groups (all *P* > 0.05). After 12 months of intervention, the intervention group exhibited significantly superior control rates for all three indicators compared to the control group.

**Table 2:** Comparison of BMI, blood pressure, and blood glucose control rates between groups.

Group	*n*	BMI controlled		Blood pressure controlled		Blood glucose controlled	
		Baseline	12 Mo	Baseline	12 Mo	Baseline	12 Mo
Control	75	26	33	27	43^a^	30	44^a^
Intervention	75	23	45^b^	29	55^b^	31	61^b^
*χ* ^2^		0.302	4.010	0.118	4.167	0.025	9.09
*P*-Value		>0.5	<0.05	>0.5	<0.05	>0.5	<0.001

a *P* < 0.05 compared with baseline.

b *P* < 0.001 compared with baseline.

**BMI Control Rate** (<24 kg/m^2^): The intervention group achieved a control rate of 60.0% (45/75), significantly higher than the control group's rate of 34.7% (26/75) (*P* < 0.05).

**Blood Pressure Control Rate** (<140/90 mmHg): The intervention group's control rate was 73.3% (55/75), significantly exceeding the control group's 57.3% (43/75) (*P* < 0.05).

**Blood Glucose Control Rate** (<6.1 mmol/L): The intervention group's control rate was 81.3% (61/75), significantly higher than the control group's 58.7% (44/75) (*P* < 0.001).

Furthermore, the intervention group demonstrated significant improvements in all target control rates from baseline to the 12-month follow-up.

### 5.3 Follow-up results of LVEF and plasma NT-proBNP

Table 3 shows baseline LVEF and plasma NT-proBNP levels were comparable between groups (all *P* > 0.05). After 12 months, the intervention group showed a significantly greater improvement in LVEF compared to the control group (52.20 ± 9.89% vs. 48.37 ± 7.05%, *P* < 0.05). No significant difference in plasma NT-proBNP levels was observed between the groups at follow-up (*P* > 0.05). The intervention group also exhibited a marked improvement in LVEF from its baseline value (*P* < 0.001), while the slight decline in mean NT-proBNP was not statistically significant (*P* > 0.05).

**Table 3:** Comparison of LVEF and plasma NT-proBNP between the control and intervention groups (mean ± SD).

Group	*N*	LVEF (%)		NT-proBNP	
		Baseline	12 Mo	Baseline	12 Mo
Control	75	45.58 ± 6.24	48.37 ± 7.05	837.31 ± 159.26	772.88 ± 189.61
Intervention	75	44.69 ± 6.27	52.20 ± 9.89^a^	819.53 ± 191.37	773.79 ± 194.95
*χ*^2^		−0.026	−2.025	0.618	−0.029
*P*-Value		>0.5	<0.05	>0.5	>0.5

a *P* < 0.001 compared with baseline.

### 5.4 Follow-up results for anticoagulant and heart rate control medication usage

Table 4 shows baseline rates of anticoagulant and heart rate control medication usage were comparable between groups (*P* > 0.05). At the 12-month follow-up, the intervention group exhibited a significantly higher rate of standardized anticoagulant therapy (74.67%, 56/75) compared to the control group (46.67%, 35/75) (*P* < 0.001). Similarly, the rate of standardized heart rate control medication usage was significantly higher in the intervention group (65.33%, 49/75) than in the control group (46.67%, 35/75) (*P* < 0.05). Both medication usage rates in the intervention group showed significant improvement from baseline (*P* < 0.01).

**Table 4:** Comparison of anticoagulant and heart rate control medication usage between groups.

Group	*n*	Anticoagulant users		Heart rate control users	
		Baseline	12 Mo	Baseline	12 Mo
Control	75	24	35	27	35
Intervention	75	22	56a	29	49a
*χ*^2^		0.125	16.477	0.118	5.247
*P*-Value		>0.5	<0.001	>0.5	<0.05

### 5.5 Follow-up endpoint events

During the follow-up period, the incidence of the primary composite endpoint (heart failure or stroke) was significantly lower in the intervention group (6.67%, 5/75) than in the control group (16.00%, 12/75) (Fisher's exact test, *P* = 0.049). For secondary endpoints, the incidence of heart failure was 5.33% (4/75) in the intervention group vs. 12.00% (9/75) in the control group (*P* = 0.154), and the incidence of stroke was 1.33% (1/75) vs. 4.00% (3/75), respectively (*P* = 0.623). No significant difference in estimated glomerular filtration rate (eGFR) was observed between the groups after 12 months.

### 5.6 Subgroup analyses

Table 5 shows the primary composite endpoint event rates, odds ratios, and interaction *P* values for the prespecified subgroups (age, sex, CHA_2_DS_2_-VASc score). No significant interactions were detected for age (*P* for interaction = 0.82), sex (*P* for interaction = 0.75), or CHA_2_DS_2_-VASc score (*P* for interaction = 0.18), indicating consistent intervention effects across all subgroups. Absolute risk reductions were numerically larger in patients aged ≥75 years (9.8% vs. 8.3%) and those with CHA_2_DS_2_-VASc ≥4 (11.6% vs. 6.3%).

**Table 5:** Intervention effects on the primary composite endpoint across prespecified subgroups.

Subgroup	Category	*n*	Control event rate (n/N)	Intervention event rate (n/N)	OR (95% CI)	*P* value	*P* for interaction
Age	<75 years	48	12.5% (3/24)	4.2% (1/24)	0.30 (0.03–3.07)	0.36	0.82
	≥75 years	102	17.6% (9/51)	7.8% (4/51)	0.40 (0.11–1.42)	0.15	
Sex	Male	72	16.2% (6/37)	8.6% (3/35)	0.49 (0.11–2.17)	0.35	0.75
	Female	78	15.8% (6/38)	5.0% (2/40)	0.28 (0.05–1.53)	0.14	
CHA_2_DS_2_-VASc	2–3	64	12.5% (4/32)	6.2% (2/32)	0.47 (0.08–2.79)	0.41	0.18
	≥4	86	18.6% (8/43)	7.0% (3/43)	0.33 (0.08–1.38)	0.13	

## 6 Discussion

Amidst population aging and the escalating burden of chronic diseases, the primary locus for atrial fibrillation (AF) management is inevitably shifting towards the community level. The model presented herein, through strategic resource integration, technological empowerment, and institutional innovation, offers a replicable solution for standardized AF management at the grassroots level.

### 6.1 Summary of principal findings and subgroup consistency

After 12 months of generalist-specialist collaborative intervention, the intervention group achieved significantly higher control rates for BMI, blood pressure, and blood glucose compared to the control group. LVEF was also markedly improved, indicating benefits in both metabolic and cardiac function. Standardized usage rates for anticoagulant and heart rate control medications were significantly higher in the intervention group, and the incidence of the primary composite endpoint (heart failure or stroke) was notably lower (6.67% vs. 16.00%). Subgroup analyses revealed no significant interactions by age, sex, or CHA_2_DS_2_-VASc score (all *P* for interaction >0.05), suggesting a consistent treatment effect across these strata and supporting broad applicability.

### 6.2 Mechanisms of comprehensive intervention effectiveness: generalist-specialist collaboration, digital tools, and comorbidity co-management

The effectiveness of this integrated management model is attributed to three main mechanisms.

First, the close collaboration between generalists and specialists creates a structured network for comprehensive care, with clear roles in treatment planning, long-term management, and patient education. Specialists formulated initial plans based on comprehensive cardiovascular risk; GPs provided long-term medication, lifestyle, and psychosocial support, leveraging trust to boost adherence; assistants ensured standardized education, data collection, and follow-up. This stratified collaboration avoids the information fragmentation of isolated specialty care.

Second, digital health tools enable real-time data sharing and risk alerts, supporting timely and precise adjustments to therapy based on key clinical indicators. AI-assisted telephone follow-up and WeChat mini-programs to achieve real-time data sharing and risk alerts. Routine monitoring of key indicators (e.g., NT-proBNP, LVEF) established a rapid response pathway (“indicator change → risk assessment → treatment adjustment”) (17–19), shifting management from empirical decision to precision intervention. For example, rising NT-proBNP triggered reassessment of blood pressure or heart failure control; declining LVEF prompted optimization of rate control or anticoagulation intensity.

Third, unified management of multiple risk factors—such as hypertension, diabetes, and obesity—helps interrupt the progression of comorbidities and enhances patient adherence through regular follow-up in the community setting. The model embedded AF care within a unified framework for hypertension, diabetes, and obesity (20), targeting upstream drivers to interrupt disease progression (5). The community setting integrated health interventions into daily life, promoting adherence and self-management. However, challenges persist in coordinated cardio-cerebrovascular management, particularly in anticoagulation decision-making and AF-specific GP training (21).

### 6.3 Secondary causes of AF and the extension of generalist-specialist collaborative management

In addition to common comorbidities, secondary causes of atrial fibrillation (AF) are important for management and prognosis. Hyperthyroidism, as a reversible cause, can be identified through routine thyroid function screening in primary care and should be integrated into the collaborative management model (22). Lifestyle factors such as excessive alcohol consumption and smoking also increase AF risk (23), and general practitioners are well-positioned to deliver sustained behavioral interventions, although this study focused mainly on general health education. The association between endurance exercise and AF, increasingly recognized in younger athletic populations (24), was not represented in the predominantly elderly cohort of this study (mean age 77.8 years). Structural heart diseases and inherited channelopathies require specialist involvement (25, 26), with generalists responsible for screening and ongoing monitoring. Secondary causes can influence stroke and bleeding risk beyond the CHA_2_DS_2_-VASc score; the collaborative framework integrates GPs' longitudinal observations with specialists' assessment of the AF substrate to support individualized anticoagulation (21). Future models should incorporate systematic screening for reversible causes (thyroid function testing, echocardiography) as a standard component of initial community evaluation.

Furthermore, cardiac implantable electronic devices (CIEDs) and home monitoring are increasingly valuable for AF detection. Subclinical AF, presenting as atrial high-rate episodes, elevates ischemic stroke risk (27). Integrating CIED-based rhythm monitoring into this collaborative model, with remote specialist review aligned with the existing digital infrastructure and GPs coordinating on-site care, could enhance early detection and timely anticoagulation.

### 6.4 Comparison with international and domestic models and the generalizability of the present model

This model aligns with international nurse-led and primary care–oriented AF management practices (9, 28), while addressing gaps in domestic literature regarding standardized workflows and integrated cardio-cerebrovascular care (29). It was implemented within China's three-tiered healthcare system, leveraging features such as Shanghai's mature family doctor contract system (30), healthcare assistants, and digital health tools. Despite some context-specific elements, the core principles—structured care pathways, clear generalist-specialist roles, protocol-driven risk factor management, and technology-enhanced patient engagement—are transferable across healthcare systems. Subgroup analysis confirmed consistent intervention effects across age, sex, and CHA_2_DS_2_-VASc strata, supporting broad generalizability. This is consistent with the ALL-IN trial (31), while differences from the mAFA-II trial may be due to sample size and statistical power. our results nonetheless extend integrated AF care evidence in primary care, especially in super-aging societies. International GP-led and nurse-led AF management programs have shown similar benefits (9, 28). The unique aspect of this model is its focus on multi-risk-factor co-management within a structured generalist-specialist collaborative framework, rather than nurse-delegated or specialist-only follow-up. Adaptation requires attention to local primary care capacity, referral infrastructure, digital health readiness, and healthcare financing.

## 7 Study limitations

This study has several limitations. First, the single urban community cohort limits generalizability. Second, the 12-month follow-up captures only short-term outcomes and cannot evaluate long-term events such as recurrent stroke, heart failure rehospitalization, or mortality. Third, the multi-component intervention precludes isolation of individual element effects. Future studies should adopt multicenter, large-sample designs with extended follow-up, incorporate component efficacy analyses and health economic evaluations, and integrate systematic screening for reversible AF causes into initial community-based assessment. Policy efforts should promote access to anticoagulation monitoring, enhance AF-specific GP training, and innovate payment models to facilitate broader adoption of this integrated community-based approach.

## Data Availability

The raw data supporting the conclusions of this article will be made available by the authors, without undue reservation.
